# Supplementary material for: Role of Enzymic Antioxidants in Mediating Oxidative Stress and Contrasting Wound Healing Capabilities in Oral Mucosal/Skin Fibroblasts and Tissues
Source: Antioxidants (Basel). 2023 Jun 30;12(7):1374. doi: 10.3390/antiox12071374 (PMC10375950; doi:10.3390/antiox12071374)
Supplement: Supplementary file 1 [file antioxidants-12-01374-s001.zip › antioxidants-2483160-supplementary.pdf]

Supplementary Figure S1

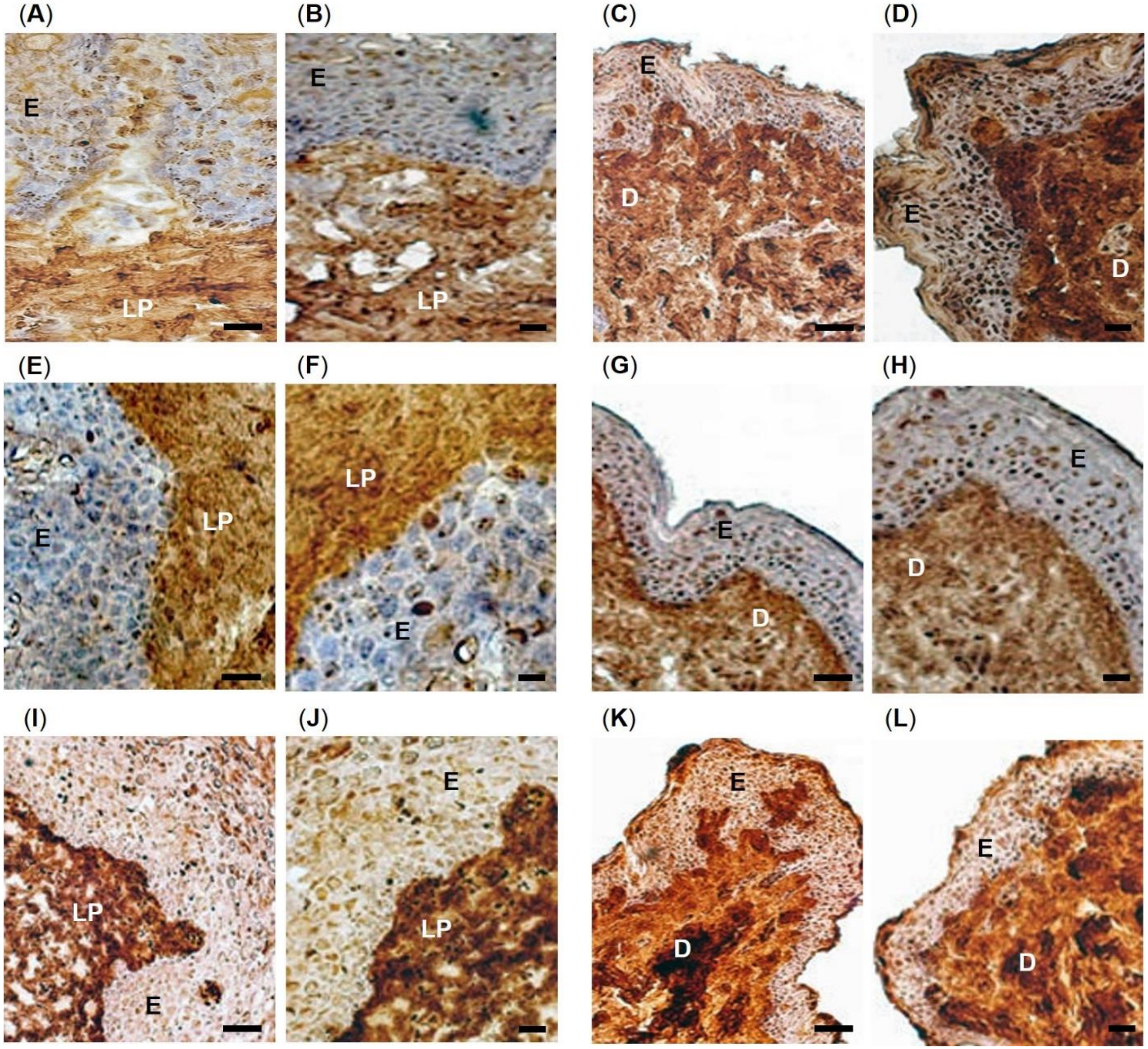

Immunohistochemical localization of protein carbonyl content in patient-matched oral mucosal and skin tissues. Low and high magnification images of (**A-B**) oral mucosal and (**C-D**) skin tissues from patient 2. Low and high magnification images of (**E-F**) oral mucosal and (**G-H**) skin tissues from patient 3. Low and high magnification images of (**I-J**) oral mucosal and (**K-L**) skin tissues from patient 4. E= Oral mucosal epithelium or skin epidermis; LP = oral mucosal lamina propria; D = skin dermis. Scale bars = 200 µm and 25 µm, respectively.

Supplementary Figure S2

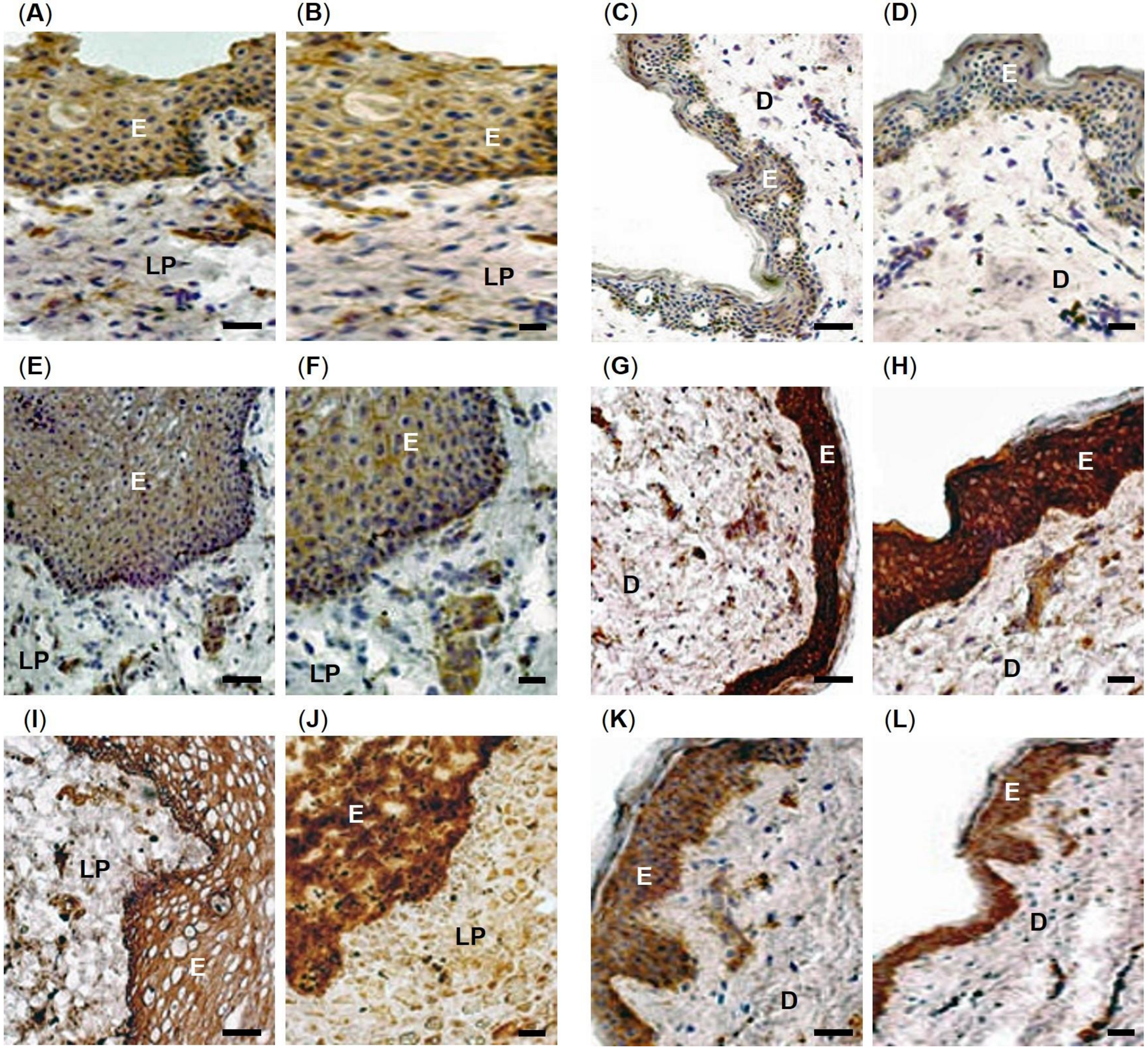

Immunohistochemical localization of malondialdehyde content in patient-matched oral mucosal and skin tissues. Low and high magnification images of (**A-B**) oral mucosal and (**C-D**) skin tissues from patient 2. Low and high magnification images of (**E-F**) oral mucosal and (**G-H**) skin tissues from patient 3. Low and high magnification images of (**I-J**) oral mucosal and (**K-L**) skin tissues from patient 4. E= Oral mucosal epithelium or skin epidermis; LP = oral mucosal lamina propria; D = skin dermis. Scale bars = 200 µm and 25 µm, respectively.

Supplementary Figure S3

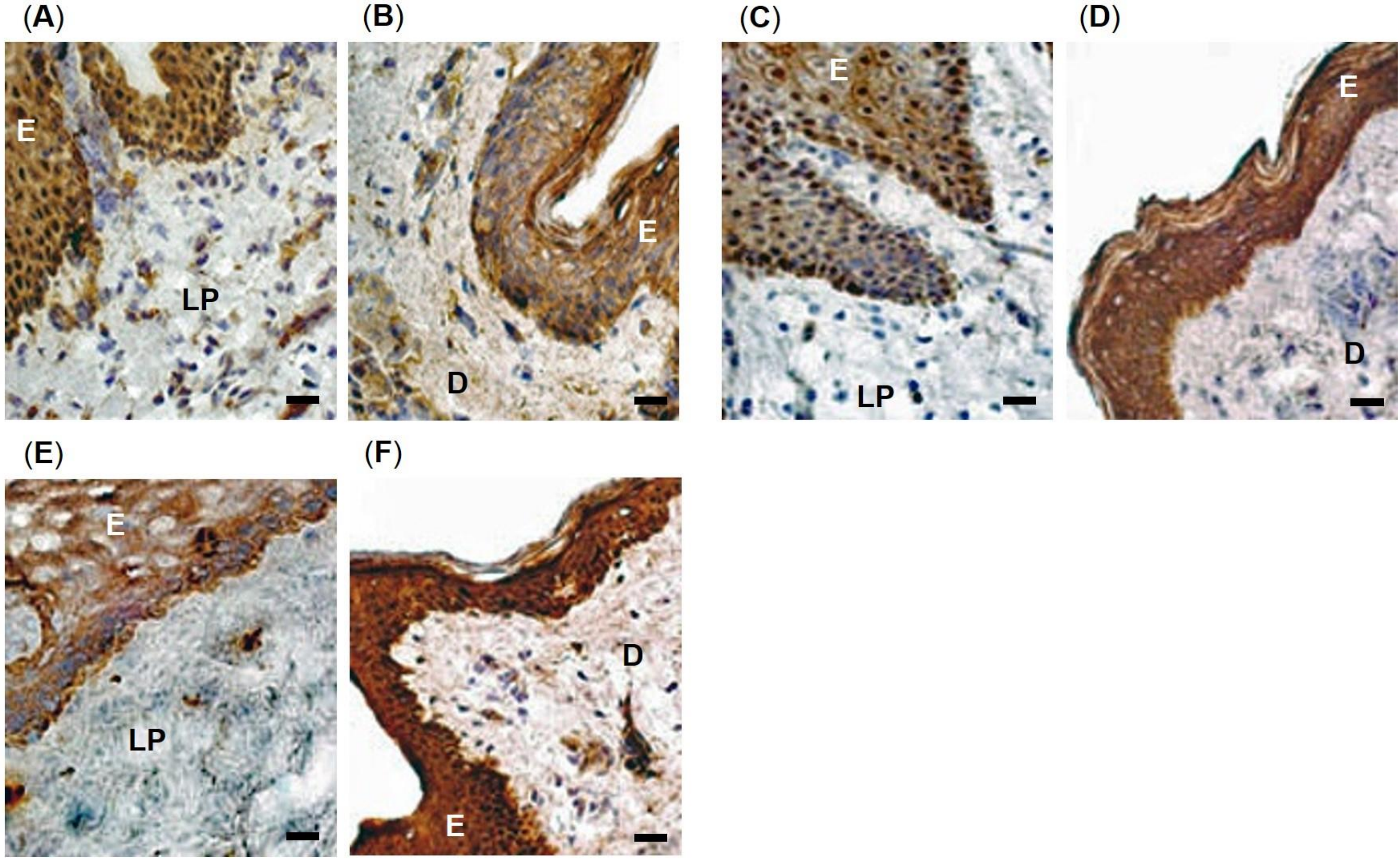

Immunohistochemical localization of SOD1 content in patient-matched oral mucosal and skin tissues. High magnification images of (**A**) oral mucosal and (**B**) skin tissues from patient 1. High magnification images of (**C**) oral mucosal and (**D**) skin tissues from patient 3. High magnification images of (**E**) oral mucosal and (**F**) skin tissues from patient 4. E= Oral mucosal epithelium or skin epidermis; LP = oral mucosal lamina propria; D = skin dermis. Scale bar = 25 μm.

Supplementary Figure S4

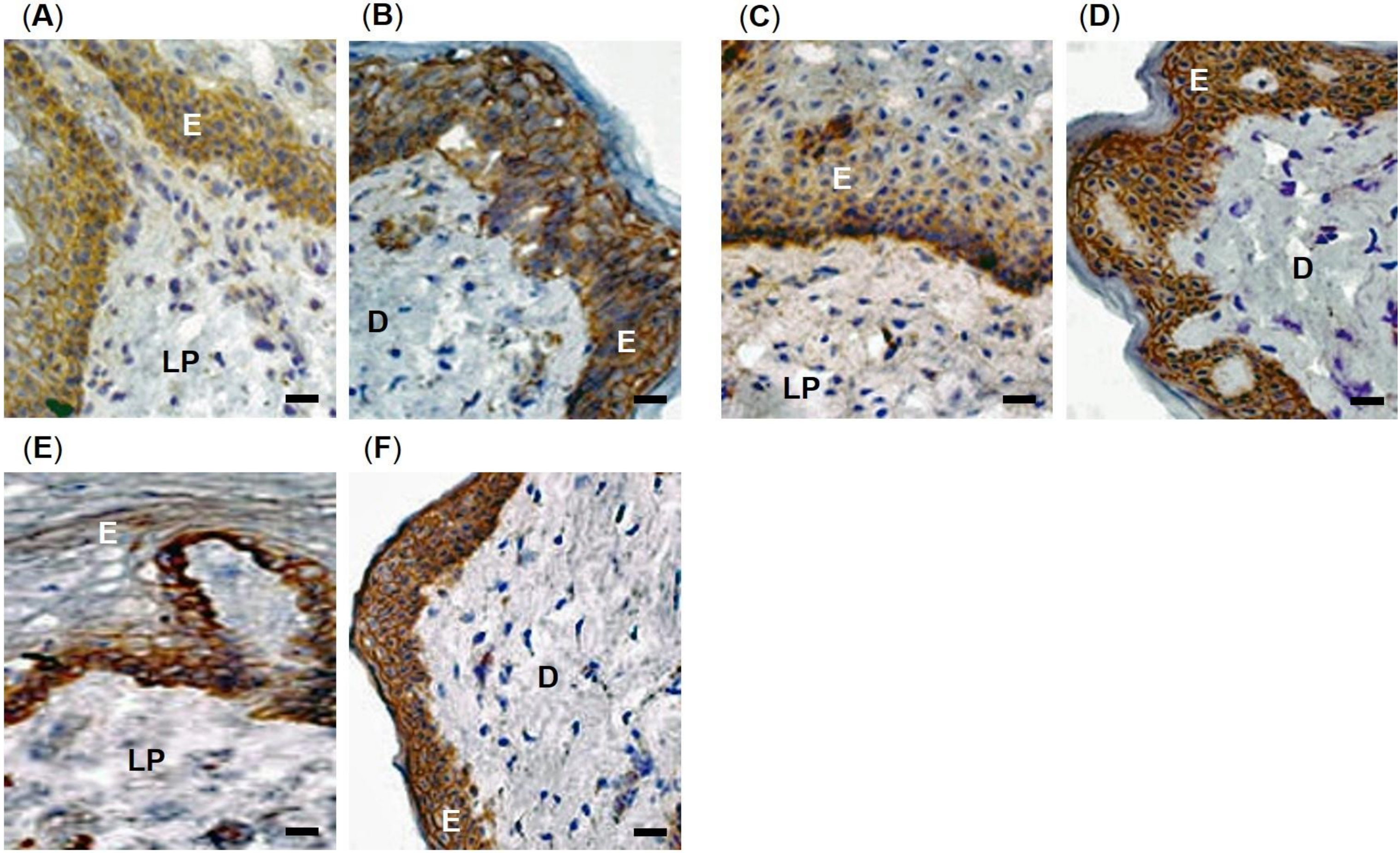

Immunohistochemical localization of SOD2 content in patient-matched oral mucosal and skin tissues. High magnification images of (**A**) oral mucosal and (**B**) skin tissues from patient 1. High magnification images of (**C**) oral mucosal and (**D**) skin tissues from patient 2. High magnification images of (**E**) oral mucosal and (**F**) skin tissues from patient 4. E= Oral mucosal epithelium or skin epidermis; LP = oral mucosal lamina propria; D = skin dermis. Scale bar = 25 μm.

Supplementary Figure S5

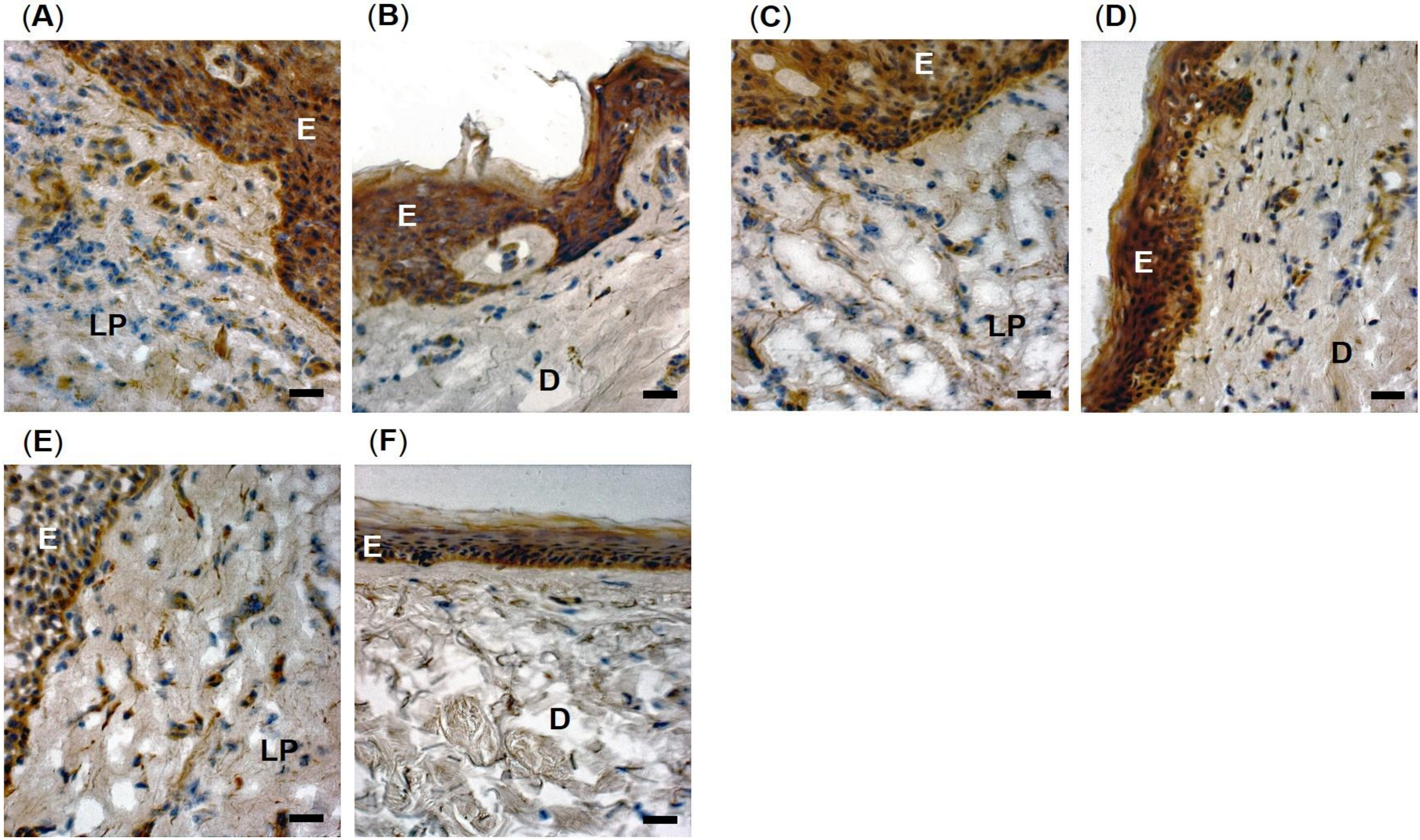

Immunohistochemical localization of SOD3 content in patient-matched oral mucosal and skin tissues. High magnification images of (**A**) oral mucosal and (**B**) skin tissues from patient 1. High magnification images of (**C**) oral mucosal and (**D**) skin tissues from patient 2. High magnification images of (**E**) oral mucosal and (**F**) skin tissues from patient 3. E= Oral mucosal epithelium or skin epidermis; LP = oral mucosal lamina propria; D = skin dermis. Scale bar = 25 μm.

Supplementary Figure S6

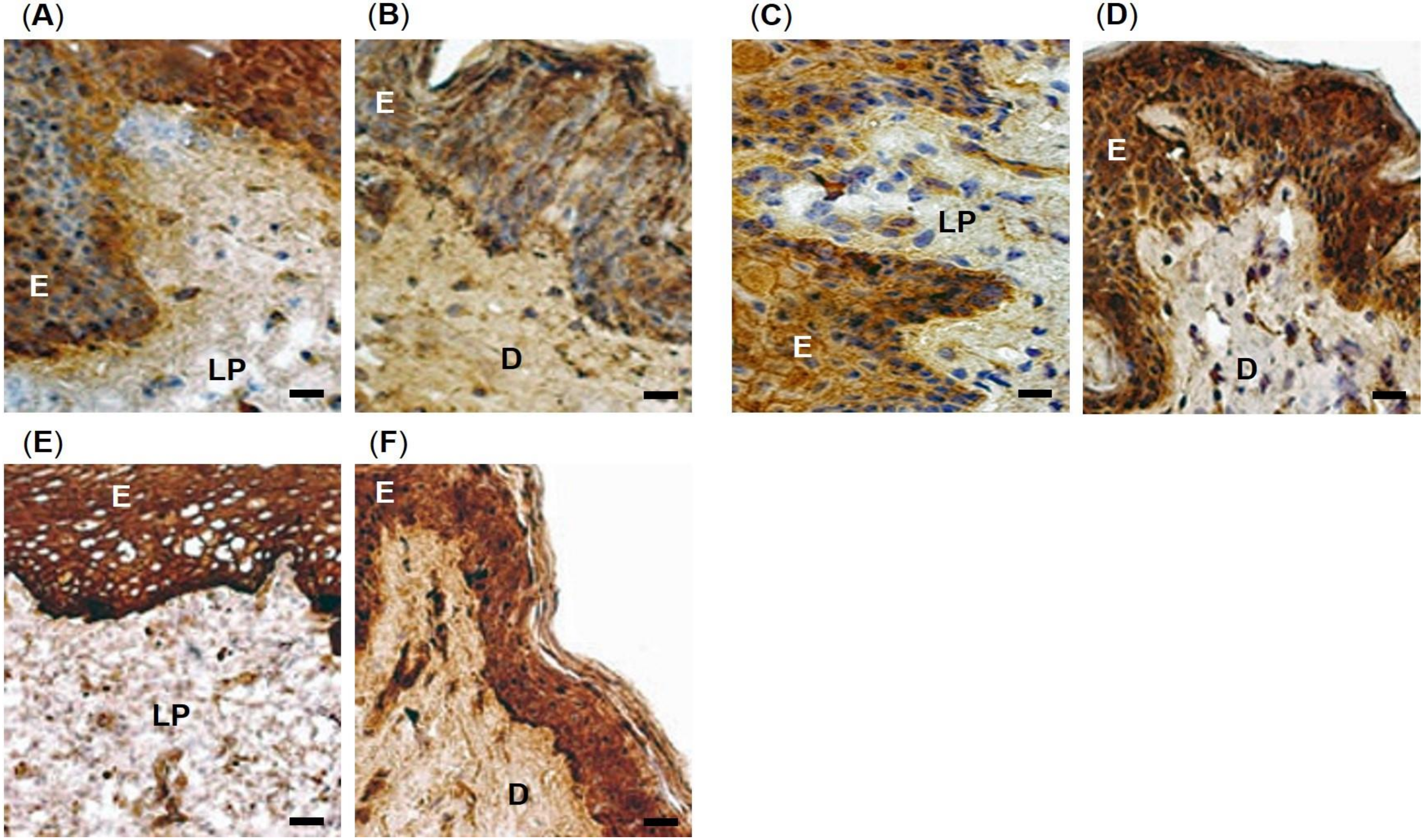

Immunohistochemical localization of catalase content in patient-matched oral mucosal and skin tissues. High magnification images of (**A**) oral mucosal and (**B**) skin tissues from patient 1. High magnification images of (**C**) oral mucosal and (**D**) skin tissues from patient 2. High magnification images of (**E**) oral mucosal and (**F**) skin tissues from patient 4. E= Oral mucosal epithelium or skin epidermis; LP = oral mucosal lamina propria; D = skin dermis. Scale bar = 25 μm.

Supplementary Figure S7

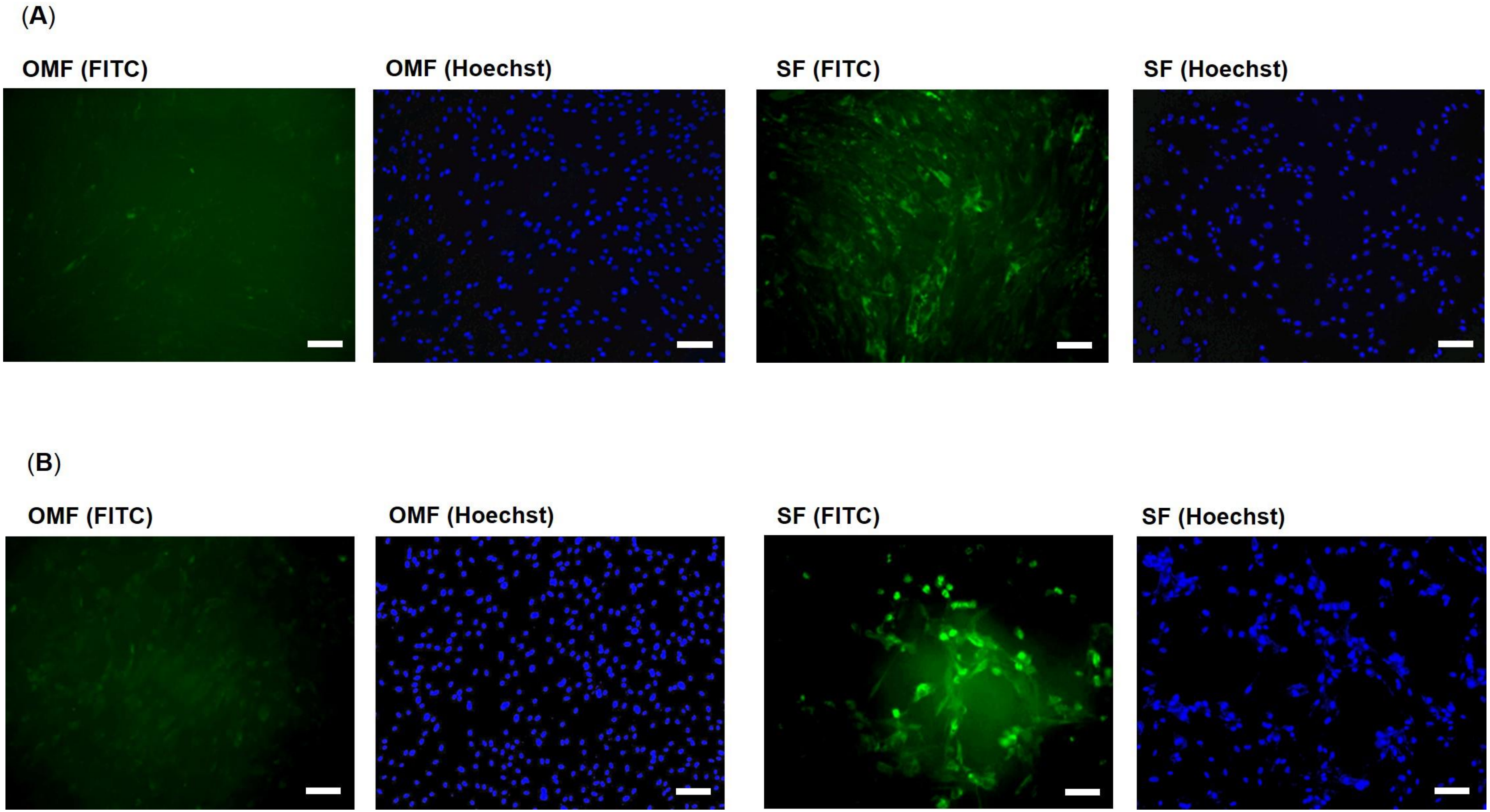

FITC (*green*) and Hoechst nuclear stain (*blue*) fluorescence microscopy images of DCF detection and ROS generation by patient- and passage-matched OMFs and SFs from (**A**) patient 6 and (**B**) patient 7. Scale bar = 200 μm.

Supplementary Figure S8

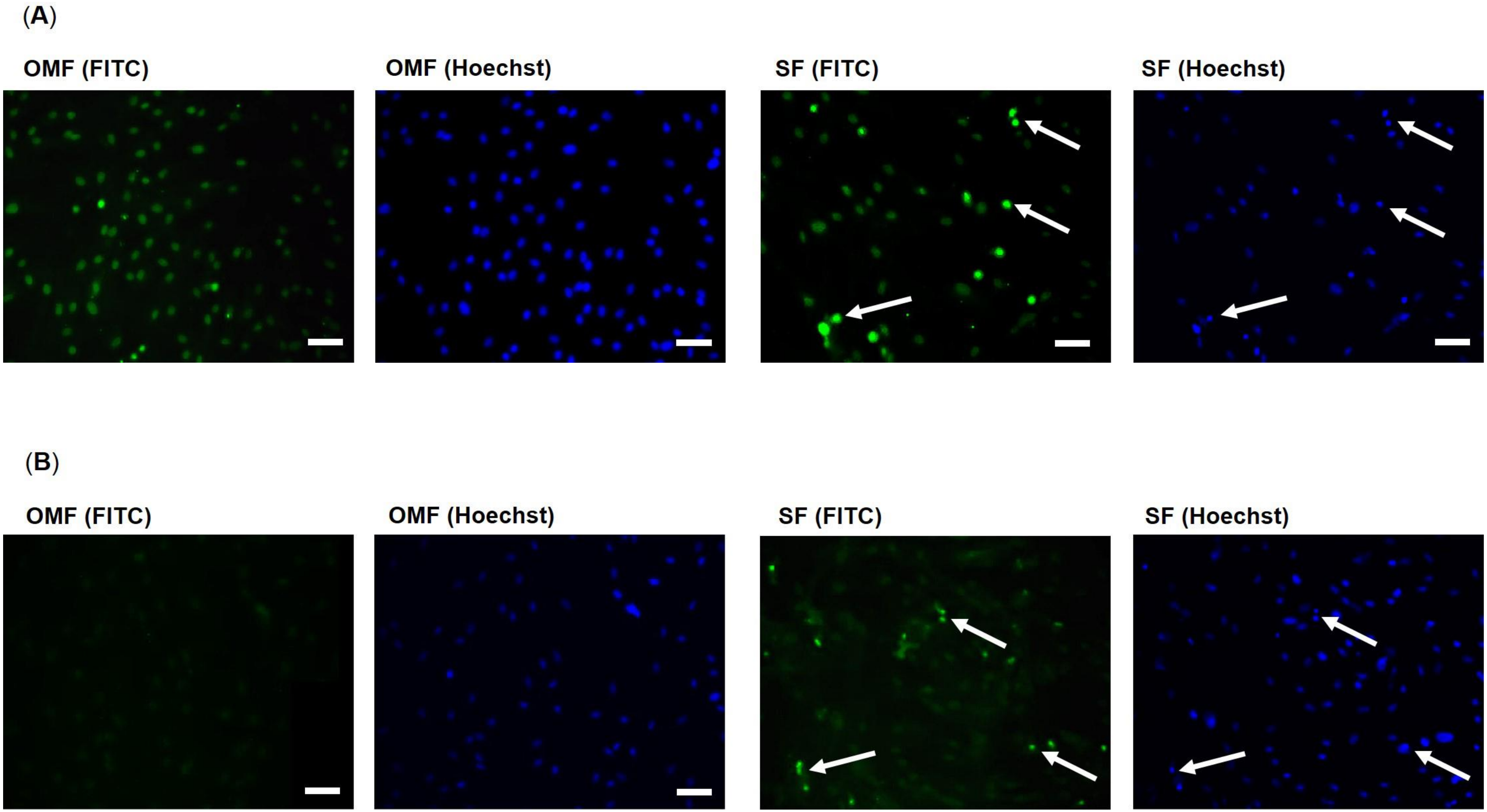

FITC (*green*) and Hoechst nuclear stain (*blue*) fluorescence microscopy images of 8-OHdG detection and oxidative DNA damage (*arrowed*) in patient- and passage-matched OMFs and SFs from (**A**) patient 6 and (**B**) patient 7. Scale bar = 100 μm.

Supplementary Figure S9

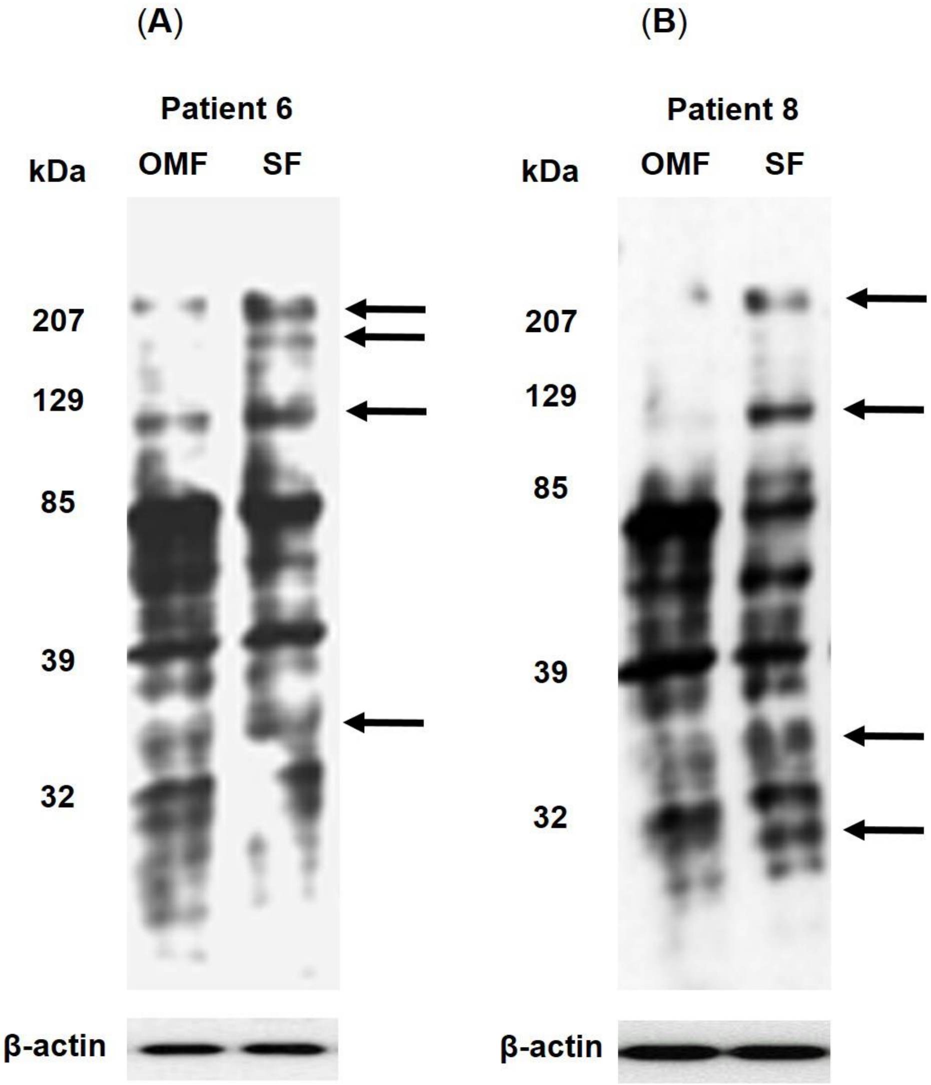

Western blot profiles of protein carbonyl content and oxidative protein damage in patient- and passage-matched OMFs and SFs from patients 6 and 8. Prominent protein carbonyl bands (*arrowed*) consistently detectable at elevated levels in SF blots are shown. For patients 6, prominent bands were detectable in SF blots at molecular weights of approximately >200 kDa, 190 kDa, 129 kDa, and 30-37 kDa (*arrowed*). For patient 8, prominent bands were detectable in SF blots at molecular weights of approximately >200 kDa, 129 kDa, and 30-37 kDa (*arrowed*).
